# A Case of a Large Peritonsillar Abscess

**DOI:** 10.7759/cureus.100199

**Published:** 2025-12-27

**Authors:** Sean S Jacob, Sinoj K John

**Affiliations:** 1 School of Medicine, University of South Carolina, Columbia, USA; 2 Division of Critical Care, Department of Medicine, Halifax Health, Daytona Beach, USA

**Keywords:** acute airway obstruction, emergency tracheostomy, parapharyngeal abscess, peritonsillar abscess, stridor

## Abstract

A patient with a peritonsillar abscess (PTA) may initially appear clinically stable, but certain risk factors can predispose to a sudden airway obstruction. Those risk factors include being a young-to-middle-aged male, obesity (defined as a BMI ≥30.0 kg/m²), or diabetes. An obese 52-year-old diabetic male presented to the emergency department with throat pain, trismus, and a muffled voice. CT imaging of the neck without contrast showed a large abscess extending into the parapharyngeal space and compressing the airway anteriorly. Although he initially had normal oxygenation, he suddenly lost consciousness and experienced cardiac arrest during a positional change during the gurney-to-bed transfer. Intubation was not possible due to mechanical obstruction, and a percutaneous tracheostomy was performed during chest compressions. Return of spontaneous circulation was achieved, and the patient recovered without neurological deficits. This case demonstrates that patients with PTA and certain comorbidities may suddenly deteriorate, even when they initially appear clinically stable. Early airway management is essential when the risk factors are present.

## Introduction

Peritonsillar abscess (PTA) is a collection of pus in the peritonsillar space [1]. It is a deep neck infection that is typically managed with antibiotics and surgical drainage [2]. In rare situations, it can rapidly become life-threatening. We present the case of a 52-year-old man whose PTA caused an acute, complete airway obstruction and subsequent cardiac arrest within minutes. This case points out the unpredictability of PTA and the critical importance of early airway management, clinical vigilance, and rapid intervention. We outline the warning signs and decision points that can help emergency providers identify patients at risk and act before decompensation occurs.

## Case presentation

**Figure 1:**
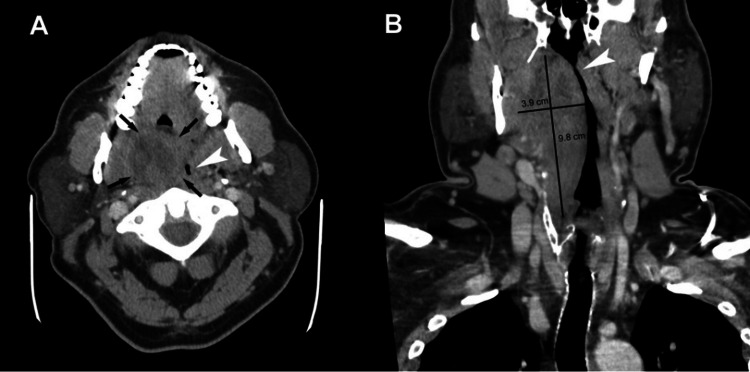
(A) shows the axial view with peritonsillar abscess (black arrows denote the margin) compressing the airway (white arrowhead). (B) shows the coronal view of the peritonsillar abscess (marked 9.8 × 3.8 cm) extending to the larynx. Note the narrowing of the airway (white arrowhead) by the large abscess.

A 52-year-old Caucasian male with a past medical history of type 2 diabetes and obesity (defined as a BMI ≥30.0 kg/m²) presented to the emergency department with throat pain, trismus, dysphagia, and dysphonia. He had streptococcal pharyngitis and was on antibiotics for one week prior to the presentation. On arrival, he appeared ill and febrile, with leukocytosis (WBC 17.1 ×10⁹/L) and a marked left shift (neutrophils 87.2%) (Table 1). A neck CT revealed a large right-sided peritonsillar/parapharyngeal abscess (3.9 × 3.7 × 9.8 cm) extending to the larynx and pyriform sinuses, causing severe airway narrowing (Figure 1). He was started on IV dexamethasone and ampicillin/sulbactam, while otolaryngology was consulted. He was initially stable with adequate oxygenation, but he suddenly developed stridor, respiratory distress, and cardiac arrest during the transfer from the gurney to the bed. CPR was started immediately. Several attempts at fiberoptic nasal and oral intubation were unsuccessful due to mechanical airway obstruction from the abscess. With compressions ongoing, an emergency percutaneous tracheostomy was performed, and placement was confirmed by fiberoptic bronchoscopy. The patient experienced cardiopulmonary arrest from severe hypoxia. ACLS protocol was followed with immediate CPR. Emergency bedside tracheostomy was performed, and once the airway was established, the patient had a return of spontaneous circulation. Total CPR time was nine minutes. The patient was admitted to the intensive care unit for airway monitoring and post-resuscitation care. Neurological status was assessed clinically following return of spontaneous circulation and demonstrated no focal deficits or cognitive impairment; neuroimaging was not indicated. The patient was subsequently extubated and decannulated following clinical stabilization.

**Table 1:** Lab findings ALT, alanine aminotransferase; AST, aspartate aminotransferase; BUN, blood urea nitrogen; ESR, erythrocyte sedimentation rate; MCV, mean corpuscular volume

Test	Pre-event	Post-event	Normal Range
WBC	17.1	13	4.0-11.0 × 10³/µL
RBC	4.53	3.64	4.5-5.9 × 10⁶/µL
Hemoglobin	14.9	12.4	13.0-17.0 g/dL
Hematocrit	43.5	36.8	39-51%
MCV	96	101.2	80-100 fL
Platelets	227	231	150-450 × 10³/µL
Sodium	134	145	136-145 mEq/L
Potassium	3.5	3.7	3.5-5.1 mEq/L
Chloride	104	115	98-107 mEq/L
BUN	20	28	7-18 mg/dL
Creatinine	0.93	1.13	0.6-1.3 mg/dL
Random glucose	156	114	74-106 mg/dL
Calcium	9.9	8.4	8.5-10.1 mg/dL
Magnesium	2.7	2.7	1.5-2.5 mg/dL
AST	68	72	15-37 U/L
ALT	88	92	12-78 U/L
Troponin I	-	404	0.0-79 pg/mL
Albumin	3.7	2.8	3.4-5.0 g/dL
ESR	77	77	0-20 mm/hour

## Discussion

PTA has established treatment protocols, but this case shows the often overlooked risk: rapid, position-dependent airway obstruction. Emergency cricothyroidotomy (ECT) may create a working channel to maintain ventilation; however, it has severe risks and must be performed by an experienced physician [3]. An ECT must be converted to a tracheostomy to prevent subglottic stenosis; this may put the patient at risk of airway compromise again [4]. PTA-related airway obstructions are more common in teenagers and young adults, with case data showing 62% male representation and a median age of 17 [5]. Comorbidities like smoking, diabetes, and obesity further add to the risk. The pattern suggests that young to middle-aged men presenting with PTA should be handled with caution, because this group may be more prone to rapid decompensation, thus airway planning should begin early. Our patient, despite appearing stable, went into sudden cardiopulmonary arrest with only a slight change in neck position. The main lesson is that a stable appearance can be misleading. While fiberoptic intubation is the go-to method for difficult airways, it may be unsuccessful in these situations because of distorted anatomy or a lack of time. Inability to safely intubate leaves a surgical airway as the only remaining option. Patients with obesity or diabetes are especially vulnerable, as obesity is associated with challenging airway anatomy, while diabetes increases susceptibility to infection and more severe PTA formation, increasing the risk of rapid clinical deterioration. Closer monitoring and earlier airway planning are necessary for them.

## Conclusions

This case challenges the traditional mindset in the management of PTA. It should be considered not only as an infection, but as a potential airway emergency well before a patient decompensates. Early risk recognition is needed for patients with distorted anatomy or comorbidities. Most importantly, the safest method is often the one planned before a crisis hits.
